# Acknowledgment to Reviewers of *Pathogens* in 2021

**DOI:** 10.3390/pathogens11020189

**Published:** 2022-01-30

**Authors:** Pathogens Editorial Office

**Affiliations:** MDPI AG, St. Alban-Anlage 66, 4052 Basel, Switzerland

Rigorous peer-reviews are the basis of high-quality academic publishing. Thanks to the great efforts of our reviewers, *Pathogens* was able to maintain its standards for the high quality of its published papers. Thanks to the contribution of our reviewers, in 2021, the median time to first decision was 16 days, and the median time to publication was 38 days. The editors would like to extend their gratitude and recognition to the following reviewers for their precious time and dedication, regardless of whether the papers they reviewed were finally published:
Abatih, EmmanuelLee, JustinAbbadi, MiriamLee, KippeumAbdad, Mohammad YazidLee, SangmiAbdela, WoubitLee, Seung-HunAbdel-Azeem, Ahmed MohamedLee, Seung-JinAbdelfattah, EssamLee, Sonny T. M.Abdel-Glil, Mostafa Y.Legaz, IsabelAbd-Elsalam, KamelLeggett, BarbaraAbhishek, SudhanshuLeggewie, MaykeAbnave, PrasadLegione, AlistairAbuley, IsaacLehner, AngelikaAcharya, ArpanLeitsch, DavidAdadi, PariseLenz, Laurel L.Adam, NovobilskýLeroux, Louis-PhilippeAdams, Sandra D.Letek, MichalAdamus-Białek, WiolettaLetoha, TamásAdhikari, RajanLetra Mateus, TeresaAguilar, Xavier FernándezLevano, KellyAhmad Khan, FaisalLewis, Leslie C.Ahmad, AsrarLeyson, ChristinaAitken-Palmer, CopperLi, DongmeiAkad, FouadLi, GangAkbar, Sheikh Mohammad FazleLi, GuangyuAkio, NakaneLi, HaoAl-Ahmad, AliLi, JixuAlberola, JuanLi, Melody HingAlberto, Cornet GomezLi, TieshiAlbornoz, AmelinaLiang, KaixinAlbrecht, DavidLieber-Tenorio, ElisabethAlcover Amengual, M. MagdalenaLiechti, George W.Alex, AnoopLim, Siew PhengAlhamdan, FahdLin, Chang-ChiAl-Hasan, Majdi N.Lin, Chao-NanAliota, MatthewLin, Chuen-FuAlkassab, AbdulrahimLin, Chun-MingAllan, FionaLin, Jin-DingAllegretti, Silmara MarquesLin, Marco Yung-ChengAllen, AdrianLin, ShaoliAlonso-Monge, RebecaLin, Shih-ChaoAltamura, LouisLIN, XionghaoAlvarez, DiegoLin, Yuh-YihÁlvarez-Mercado, Ana I.Linaldeddu, Benedetto T.Alves, DianaLindhal, JohannaAlvisi, GualtieroLindhout, DickAlzate, Juan F.Linke, Reinhold P.Amagliani, GiuliaLinkies, AdaAmarilla, Alberto A.Linz, BodoAmaro, FranciscoLipari, DarioAmendola, AlessandraLipke, PeterAmimo, Joshua OluochLittle, ChrisAnastasiou, Olympia E.Liu, Chen-HuaAncuceanu, RobertLiu, Cheuk LunAndersson, Maria A.Liu, HejunAndré, Marcos RogérioLiu, ZhuomingAndréani, JulienLizano, MarcelaAndreani, Nadia AndreaLledó, LourdesAndrophy, Elliot J.Lleo, MarAndrzejczuk, SylwiaLloyd, MeganAngelici, Maria CristinaLloyd, VettAnisimov, Andrey P.Lo Cigno, IreneAntigua, Khristine Joy C.Lo, Shih-YenAntolová, DanielaLodge, Katharine M.Antunes, JoanaLodmell, J. StephenAnwar, FirozLohia, PrateekApperson, Charles S.Lojkić, IvanaArango Duque, GuillermoLoncaric, IgorArastehfar, AmirLondono, BerlinAraujo, Jackson V.Long, Carrie MaeArez, Ana PaulaLongeri, MariaArismendi, NolbertoLopes, Ana Patrícia AntunesArivett, BrockLópez-Abán, JulioArmbruster, Chelsie E.Lorberboum-Galski, HayaArsuaga, MartaLorenzo, MicheleArteaga-Livias, KovyLorenzo-Morales, JacobArukha, Ananta PrasadLove, Melissa S.Asada, MasahitoLovec, VesnaAsbach, BenediktLowry, KymAscough, StephanieLu, MaolinAsensi, VictorLucas, Eric R.Ashrafi, RoghaiehLuchian, IonutAsim, MuhammadLudwig-Słomczyńska, Agnieszka H.Asnicar, FrancescoLuo, MaAssy, NimerLuridiana, SebastianoAthanasiou, Labrini V.Luther King, Abia AkebeAtkins, Clarke E.Lütticken, RudolfAto, ManabuLynggaard, Charlotte DuchAttanayake, Renuka N.Lynn, GeoffreyAttia, Youssef A.Lyu, Jae IlAttri, KuldeepMa, JincaiAuguste, Albert J.Maboni, GrazieliAugustine, Swinburne A. J.Macaluso, KevinAugusto, LeonardoMacey, MichaelAung, Meiji SoeMachado, Luis FernandoAutier, BriceMaciver, SutherlandAvdic, SelmirMackenstedt, UteAvram, FlorinMackenzie, CharlesAwasthi, MayankaMacLeod, Ewan ThomasAzar, Sasha R.Macori, GuerrinoAzevedo Santos, HuarrissonMadeira De Carvalho, Luis ManuelAzinheira, Helena G.Maekawa, ShunBabiuk, ShawnMagagnoli, FedericaBackhouse, DavidMagariños Ferro, BeatrizBadali, HamidMagori, KrisztianBagiński, MaciejMagryś, AgnieszkaBagiu, Iulia-CristinaMahony, Timothy JohnBai, LanlanMair, KerstinBajer, AnnaMaisch, TimBaker, BrianMaisetta, GiuseppantonioBakonyi, TamásMaisse, CarineBakre, Abhijeet A.Maistrello, LaraBal, Abhijit MahendraMaiti, GeorgeBalaraman, VelmuruganMajchrzak, MartaBalczun, CarstenMajerciak, VladimirBaldin, ClaraMajewska, AnnaBaldy-Chudzik, KatarzynaMajkowska-Skrobek, GrażynaBaliraine, FrederickMajoros, GaborBalkema-Buschmann, AnneMajoros, LaszloBanadyga, LoganMajumdar, ManasiBanović, PavleMajumder, KinjalBaptista, RodrigoMak, Gannon C. K.Baranova, EkaterinaMalandrin, LaurenceBarberis, ElettraMalewski, TadeuszBarbosa, AngelaMalfertheiner, PeterBarcena, CarmenMallagaray, AlvaroBarcena, JuanMalleret, BenoitBar-Haim, ErezMallet, VincentBarker, EmiMalli Mohan, Ganesh BabuBarlaam, AlessandraMaloberti, AlessandroBarna, BalázsMaloney, JennyBarr, Kelli L.Maluquer De Motes, CarlosBarradas, Patrícia FerreiraMancianti, FrancescaBarry, GeraldMancini, LeonardoBarth, DylanManet, EvelyneBartholomeeusen, KoenManivannan, AbinayaBartholomew, JerriManuel Rodríguez Bertos, AntonioBartlow, Andrew W.Manukumar, H. M.Bártolo, InêsMarais, BenBartoloni, AlessandroMarasini, SanjayBartosik, KatarzynaMarco, AdolfoBartova, EvaMarcone, CarmineBarua, NilakshiMarfurt, JuttaBarua, SubarnaMarini, GiovanniBasco, LeonardoMarino, FabioBasheer, JasimMarino, GabrieleBasmaciyan, LouiseMarinov, M. P.Bastos Gomes, GianaMarjanović, ŽaklinaBastos, Reginaldo G.Markovska, RumyanaBates, PaulMarraccini, PaoloBatra, LalitMarruchella, GiuseppeBattilani, MaraMartin, HarleiBauza-Kaszewska, JustynaMartin, LynnBawm, SawMartin, StephenBaymakova, MagdalenaMartín, VerónicaBażanów, BarbaraMartín-Burriel, InmaculadaBazzocchi, ChiaraMartín-Cuervo, MaríaBeauclair, GuillaumeMartínez Beamonte, RobertoBeaumelle, BrunoMartinez, JuanBechara, GervasioMartínez-Carrasco Pleite, CarlosBeck, AlanMartínez-Morcillo, Francisco J.Beck, ReljaMartins, MathiasBecker, KarstenMartirosian, GayaneBedi, SukhmaniMaruyama, HaruhikoBeechler, Brianna R.Maruyama, TesshoBehnam, Mira A. M.Marzec-Schmidt, KatarzynaBehnke-Borowczyk, JolantaMas-Coma, SantiagoBejarano, AnaMasiulis, MariusBeklen, ArzuMastino, AntonioBełka, MartaMataragas, MariosBellini, SilviaMatczuk, Anna KarolinaBeltrame, AnnaMatei, IoanaBelyi, YuryMateos-Hernández, LourdesBemis, Lynne T.Matsubayashi, MakotoBencúrová, ElenaMatteucci, ClaudiaBenmerzouga, ImaanMatthiesen, SveaBennett, Richard J.Matthijs, SeverineBenschop, Kimberley S. M.Matthysse, Ann G.Bentley, Emma M.Mattila, SariBenton, AngelaMattoscio, DomenicoBentwich, ZviMaugeri, Andrea GiuseppeBeran, JiriMaurer, PatricBerbee, MaryMauricio, IsabelBergey, ChristinaMaurin, MaxBergmann, SvenMay, MeghanBeris, DespoinaMayberry, ColleenBermudez, Luiz E.Mbengue, AlassaneBermudez, MarcelMcDermott, EmilyBernardini, GiuliaMcDonnel, Samantha J.Bertelloni, FabrizioMcDougal, RebeccaBerzins, AivarsMcGregor, Bethany L.Berzosa, Pedro J.McInnes, ColinBessière, PierreMcLaughlin, JohnBette, MichaelMcNeal, Monica MaloneBeyi, Ashenafi F.Mead, Daniel G.Bhattacharyya, TapanMedina, Gisselle N.Bickel, ScottMedo, JurajBiet, FranckMelegari, GabrieleBilański, PiotrMenon, Manoj B.Bilska-Zając, EwaMercer, AndyBingham, RichardMercer, JasonBiondi, AntonioMeregildo Rodriguez, Edinson DanteBiondo, CarmeloMerino, Jose JoaquinBiswal, ManishaMertens-Scholz, KatjaBitchava, KonstantinaMesquita, JoãoBitner, AgnieszkaMessadi, LiliaBlagojevic Zagorac, GordanaMestres, ConxitaBlaise, MickaelMeto, AidaBlanco-Melo, DanielMetras, RaphaelleBlanc-Potard, AnneMeurens, FrançoisBlanda, ValeriaMeyer Sauteur, Patrick M.Blažytė-Čereškienė, LaimaMezouar, SorayaBlome, SandraMiao, QiuhongBobkova, MarinaMichaelis, MartinBocedi, AlessioMichalski, MirosławBock, C. ThomasMiele, Adriana E.Boda, FranciscMigdał, ŁukaszBody-Malapel, MathildeMigliore, SergioBoere, VannerMignolet, JohannBok, EwaMihalca, Andrei DanielBolaño Ortiz, Tomás RafaelMihelčić, MirnaBolboaca, SoranaMiklasińska-Majdanik, MariaBolshoy, AlexanderMilicevic, VesnaBonelli, PieroMiller, RachelBonetta, SaraMioni, Mateus De Souza RibeiroBonetta, SilviaMioulet, ValerieBongiorno, DafneMira, FrancescoBonilauri, PaoloMiranda, CarlaBonsembiante, FedericoMiranda, ClaudioBonvicini, FrancescaMishra, AartiBorel, NicoleMishra, DushyantBorghi, ElisaMishra, Hemant K.Boschiroli, María LauraMishra, Jay S.Bosco-Lauth, Angela M.Mishra, SanketBosio, ChrisMitarai, SatoshiBotelho, JoãoMitra, Amal K.Bourgeois, DenisMitra, AvishekBourret, TravisMittapelly, PriyankaBourret, TylerMiura, TanyaBowen, RichardMiyazawa, KohtaroBowman, CaitlynMognetti, BarbaraBozdoğan, HakanMoiseenko, Konstantin V.Bozidis, PetrosMojumdar, KamalikaBraczkowski, RyszardMoniuszko, HannaBrandt, SabineMonjarás Feria, JuliaBrassard, JulieMonokrousos, NikolaosBrassinga, Ann Karen C.Monteiro, MarioBraun, Stephen E.Monteiro, SilviaBrener, BeatrizMonteiro, SílviaBrennan, GregMontero, EstrellaBrettell, Laura E.Montoya, AnaBrewer, Matthew HomasMontoya-Alonso, J. AlbertoBrianti, EmanueleMontresor, AntonioBriles, David E.Moonga, Lavel ChinyamaBrindley, MelindaMora, DiegoBrisse, MorganMora-Montes, HectorBrito, MiguelMorchón, RodrigoBrito, Nuno V.More, SunilBritz, Margaret L.Morelli, SimoneBrizić, IlijaMoreno Gonzalez, InesBromhead, ColletteMoreno Mesonero, LauraBrooks, TimMoreno, Laura GarzaBrossay, LaurentMorente, Maria Ángeles PérezBrown, JasonMoretti, SoniaBrown, RichardMorganti, GiuliaBrugliera, LuigiaMori, MarcellaBrugman, VictorMori, ShogoBruno, Giovanni L.Moriarty, Leah F.Bruschi, FabrizioMorikawa, YukoBrym, PawełMorimoto, MotokoBuesa, JavierMorio, YoshiteruBukhari, ZiaMoriyama, KazuhiroBulavaitė, AistėMorka, KatarzynaBulet, PhilippeMorphew, Russell M.Bulk, EtmarMorris, FayeBulman, SimonMorris, MhairiBulmer, Mark S.Morris, Stephen A.Buonsenso, DaniloMorroni, GianlucaBurette, MelanieMortlock, MarindaBurgara-Estrella, AlexelMorzunov, SergeyBurgess, BrandyMoseley, MarkBurke, Rachel MariaMoshammer, HannsBurkowska-But, AleksandraMosselhy, DinaBurokiene, DaivaMotamed, CyrusBurrascano, JosephMotoie, GabrielaBuschiazzo, AmbraMoura De Aguiar, DanielByrd, James AllenMourenza, ÁlvaroByrne, AndrewMoutailler, SaraCaballero, Raúl PérezMowlaboccus, ShakeelCabrefiga, JordiMrzljak, AnnaCaeiro, Maria FilomenaMugetti, DavideCalabrese, Kátia Da SilvaMukaratirwa, SamsonCalcutt, MichaelMukerji, ShibaniCaldara, MarinaMukherjee, MaitreyeeCalistri, AriannaMukherjee, ShibabrataCalla, JaesonMuller Kratz, JadelCalvani, NicholaMüller, UweCamp, Jeremy V.Munck, IsabelCampana, RaffaellaMunis, Altar M.Campbell, DavidMunita, Maria PiaCampbell, Lee AnnMunsey, AnnaCampos, Maria Jorge GeraldesMunyanduki, HenryCannon, RichardMünz, ChristianCano Calle, DanielaMurakami, KosukeCano-Terriza, DavidMurakami, ShinCantos-Barreda, AnaMurakami, TsutomuCanuti, MartaMurdaca, GiuseppeCapellari, SabinaMurillo, RosaCarcy, BernardMurphy, NiamhCardin, RhondaMurugan, RajagopalCardo, Maria VictoriaMusella, VincenzoCardoso, FernandoMutsvunguma, Lorraine Z.Cardoso, LuísMuxel, Sandra MarciaCarnaccini, SilviaMyrmel, MetteCarnell, GeorgeNa, Byoung-KukCarpentier, DavidNa, Hee SamCarr, MandiNagamori, YokoCarrillo, Catherine D.Nagle, Peter W.Carullo, GabrieleNakajima, KeiCarvalho Campos, LeilaNakao, RyoCarvalho, Carlos A. M.Nalca, AysegulCasanovas, MiguelNapoli, EttoreCascioferro, StellaNarayanan, AarthiCassini, RudiNarra, Hema PrasadCastelli, GermanoNatale, AldaCastelli, MicheleNateghpour, MehdiCastro, JoanaNavas, AlfonsoCastro, Mariana S.Navas, JesúsCaughey, ByronNebija, DashnorCavallero, SerenaNeef, AlexanderCazorla-Luna, RaúlNeely, Melody N.Cebrián Castillo, RubénNejezchlebová, HelenaCelec, PeterNeumann, DonnaCencek, TomaszNg, Wai-LungCerbo, Alessandro DiNguyen, Joe TruongČernáková, LuciaNguyen-Kim, Thi Dan LinhČerný, JiříNgwa, Che JuliusČervená, BarboraNicholas, ListerCesare, Angela DiNicoletti, LoredanaCesarini, CarlaNiendorf, SandraChae, ChanheeNiewiadomska, AlicjaChakraborty, AnirbanNiine, TarmoChakraborty, SarojNikolakakis, IoannisChambers, ThomasNikolich, MikeljonChan, MartinNikolopoulos, GeorgiosChao, Chien-MingNishi, KosukeCharco, Jorge MorenoNishide, YudaiCharvet, Claude L.Njemini, RoseChatanga, ElishaNocera, Francesca PaolaChaudhuri, MinuNock, AdamChauhan, NikhilNolan, MelissaChavanas, StéphaneNorimine, JunzoChaves, Luis FernandoNovais, ÂngelaChávez-Calvillo, GabrielaNowak-Chmura, MagdalenaChawla, Amanpreet SinghNsanzabana, ChristianChen, FengjuiNtana, FaniChen, Jiann-ChuNtanasis-Stathopoulos, IoannisChen, Pin-jungNugraha, Arifin BudimanChen, Po-LinNunes, Francis Morais FrancoChen, Yi-NingNunes, MonicaChen, Yuan-ChuanNúñez, José IgnacioCheng, Jya-WeiO′Callaghan, DavidCheng, ZishuoO′Callaghan, DennisChevrot, RomainO′Neal, HollisChilimuri, SridharOancea, FlorinChisu, ValentinaOdendaal, LiezaChiţimia-Dobler, LidiaOdoch, TerenceChitko-Mckown, CarolOgata, HiroyukiCho, Young-KeolOgata, TsuyoshiChochlakis, DimosthenisOgórek, RafałChoi, ChangsunOgunremi, DeleChoi, Kyoung SeongOishi, TomohiroChoi, Won HyungOkamoto, MarikoChomicz, LidiaOkazaki, KatsunoriChong, RogerOkodo, MitsuakiChoudhuri, SubhadipOladeinde, AdelumolaChow, Franklin Wang-NgaiOliván-Gonzalvo, G.Christodoulides, MyronOliveira, AlineChua, Eng GuanOliveira, RyanChuang, Hung-YiOlivieri, EmanuelaChuang, Wen-YuOlsen, StevenChurchward, ColinOmosun, Yusuf O.Ciancio, AurelioOng, Jason J.Cichero, ElenaOnyedibe, Kenneth I.Cichy, AnnaOrsburn, BenjaminCima-Cabal, María DoloresOrtega, Joseph ThomasCiuffo, MarinaOrtolano, SaidaClapham, HannahOrusa, RiccardoClark, CliffordOsbjer, KristinaClifford, GaryOsborne, JaneCoelho, CatarinaOscorbin, IgorCoetzer, TheresaOsorio, Elvia JanethCognasse, FabriceOsterloh, AnkeCohen, AnitaOteo, José A.Coia, JohnOtero, AliciaColdbeck-Shackley, RosaOthumpangat, SreekumarColebunders, RobertOzarowski, MarcinColeman, ChristopherPace, LorenzoColombo, MariasolePacioni, GiovanniColquhoun, IanPadmanabha Das, Krishna MohanComas-Garcia, MauricioPadungtod, PawinComer, Jason E.Pagani, LeonardoConant, KatherinePagano, MarioConceição, NatérciaPage-Karjian, AnnieConejero, LauraPaik, Soon YoungCong, YingyingPailhoriès, HélèneConlan, WaynePaillot, RomainConnelly, RoxannePajdak-Czaus, JoannaConrady, BeatePal, GargiConti, StefaniaPalacios, Oskar A.Contrant, MaudPalanisamy, NavaneethanContreras Rodríguez, AraceliPalenzuela, OswaldoCoppola, CarminePalic, DusanCork, SusanPallecchi, LuciaCorona, MiguelPalma, DanielaCorreia, InêsPalmero-Llamas, DanielCorrente, MarialauraPalomba, MarialetiziaCortegiani, AndreaPaltrinieri, SaverioCory, JennyPalusinska-Szysz, MartaCosoveanu, AndreeaPan, HongyingCosta, AdrianaPandey, SheoCosta, DamienPane, CatelloCosta-Da-Silva, André LuisPanthee, SureshCostantini, ClaudioPaoletti, BarbaraCoudray, Makella S.Papadopoulos, Elias G.Cremonesi, PaolaPapan, CihanCrocè, Lory SaveriaPaparoupa, MariaCruz, Taís FukutaPapatsiros, Vassilios G.Cuadra, GiancarloPappa, OlgaCull, BenjaminPappas, Christopher J.Cursino-Santos, Jeny R.Papp-Wallace, Krisztina M.Cuteri, VincezoParachuru, VenkataCywińska, AnnaParadzik, TinaCzubacka, AnnaParaschiv, SimonaCzyzewska-Dors, EwelinaParedes-Esquivel, Claudia CaterinaD′Agostino, DonatoParisi, FrancescaD′Angeli, FlorianaPark, ChanghoonD′Ardes, DamianoPark, Seung-ChunDąbrowska, JoannaParker, WilliamDaca, AgnieszkaParóczai, DóraDacosta, Miguel BaladoParra, GabrielDahdouh, Elias A.Parrish, ColinDahl, Jan-UlrikParsons, CameronDahle, Maria K.Partridge, LyndaDai, XinghongParvate, AmarDal Pozzo, FabianaParveen, NikhatDall Agnol, Alais MariaPascucci, IlariaDallares, SaraPasdeloup, DavidDallenga, TobiasPatil, VeerupaxagoudaDaly, JanetPatil, Vinod VikasDaly, Seth M.Patrick, S. GellingsDangi, TanushreePatterson, StevenDaniel, Carlton Ralph C.Patterson-West, JenniferDanišová, OľgaPaulitz, TimothyDarre, Michael J.Pawełczyk, OlgaDas, ShubhagataPaz-Silva, AdolfoDavid, Daniel O.Peersen, OlveDavid, JohnPeeters, MartineDavidson, Silas A.Pellegrino, IreneDavies, CarolinaPellicano, RinaldoDavis, A. SallyPellon, AizeDavis, ChelseaPelloux, HervéDavis, David A.Penagos-Tabares, FelipeDe Benedictis, PaolaPeng, DuoDe Bernard, MarinaPennance, TomDe Bonis, SalvatorePennisi, Maria GraziaDe Carolis, ElenaPeper, Steven T.De Castro, Alessandra Marnie Martins GomesPépin, MichelDe Conto, FloraPerec-Matysiak, AgnieszkaDe Curtis, FilippoPerego, RobertaDe Francesco, MariaPereira, Richard Van VleckDe Groof, AdPerelomov, Leonid V.De Groot, Piet W. J.Perez Jimenez, Jèsus MariaDe Jong, AukePérez Ramírez, ElisaDe La Maza, Luis M.Pérez-Martínez, SandraDe La Rua Domenech, RicardoPérez-Sánchez, RicardoDe la Torre, Juan C.Pericas, Juan ManuelDe Lagarde, MaudPerrucci, StefaniaDe Liberato, ClaudioPersson, KristinaDe Lima, Valéria Marçal FelixPetca, RǎzvanDe Los Santos, TeresaPeters, JonathanDe Luca, ElianaPeters, ThomasDe Marco, FedericoPeterson, Jennifer K.De Paola, DomenicoPetinaki, EfthimiaDe Swart, RikPetrone, AlessioDea-Ayuela, María AuxiliadoraPetrovan, VladDeak, GeorgianaPfaender, StephanieDearborn, Altaira D.Pfeifer, YvonneDeb, SubrataPhillips, SamuelDebnath, AnjanPhulera, SwastikDecker, Sebastian O.Piacenza, ElenaDedousi, AnnaPia̧tek, Rafał J.Deere, JessePierangeli, AlessandraDeim, ZoltánPietruczuk-Padzik, AnnaDekhnich, AndreyPignataro, GiuliaDeksne, GunitaPike, AndrewDel Real, GustavoPincus, SethDelbecq, StéphanePinheiro, JorgeDelghandi, MohammadPinto, BarbaraDeligianni, ElenaPinto, Hudson AlvesDell′Oste, ValentinaPisarenko, SergeyDellamaria, DeboraPitesky, Maurice E.Delviks-Frankenberry, KristaPittau, MarcoDemers-Mathieu, VeroniquePituch, Hanna M.Demetris, AvraamPlacido, AntonioDenagamage, ThomasPlatonov, Alexander E.Denning, David W.Platt-Samoraj, AleksandraDenzin, NicolaiPlayford, MattDeplazes, PeterPleckaityte, MildaDerdáková, MarkétaPobiega, KatarzynaDesdouits, MarionPocchiari, MaurizioDeshpande, AlinaPoirel, LaurentDeshpande, GauraviPolak, Mirosław PawełDesnos-Ollivier, MariePolat, MeripetDessau, RamPolischuk, PavelDessens, JohannesPoma, AdolfoDevasundaram, SanthiPomara, CristoforoDeviatkin, Andrei A.Pomari, ElenaDevoy-Keegan, JasonPombinho, RitaDezsényi, BalázsPomilio, FrancescoDhamotharan, KannimuthuPonsero, Alise J.Dhinakarasamy, InbakandanPopescu, Costin-IoanDi Bella, SantinaPopescu, SilvanaDi Bonito, PaolaPosautz, AnnikaDi Carlo, PaolaPoudel, AnilDi Cesare, AngelaPoupard, PascalDi Francesco, AntoniettaPowell, Daniel A.Di Gennaro, FrancescoPrasad, AbhishekDi Marco, GabrielePratt, JimDiack, Abigail B.Press, Charles McLeanDiakou, AnastasiaPreziuso, SilviaDias Junior, Antonio GregorioPrice, PatriciaDiaz Fernadez, PabloPrigigallo, Maria IsabellaDíaz-Rosales, PatríciaPrigione, ValeriaDíaz-Sánchez, Adrian AlbertoPrimavilla, SaraDietrich, IsabellePrincipe, LuigiDíez-Fernández, AlazneProft, ThomasDillman, AdlerPronin, Alexander V.DiMaio, DanielProroga, Yolande Thérèse RoseDimitrov, DimitarPuchkov, EvgenyDimov, SvetoslavPujadas, ElisabetDinman, JohnatanPujol, Flor H.Dix, RichardPulliainen, ArtoDługosz, EwaPuray-Chavez, Maritza N.Dłużniewska, JoannaPurkrtova, SabinaDobeš, PavelQiu, YongjinDobler, GerhardQualls, WhitneyDoceul, VirginieQuayle, Alison J.Doddapattar, PrakashQueiroz, Luzia HelenaDolci, MarcoQuilez, JoaquinDoligalska, MariaQuintas, HelderDolka, IzabellaRachel, GilroyDomańska-Blicharz, KatarzynaRadcliff, Fiona JaneDomingo, Gonzalo J.Radim, DobiášDominguez, Juan CarlosRadzevičienė, AurelijaDonaldson, JanetRagan, IzabelaDong, ShengzhangRahman, AyeshaDonnelly, Callum GeorgeRahman, HadiarDonnelly, SheilaRahman, Masmudur M.Dora, DavidRai, RikkyDors, ArkadiuszRaidal, Shane R.Dos Santos Dias, LucasRajasekharan, Satish KumarDouda, OndřejRajeev, SreekumariDrahovská, HanaRajeeve, KarthikaDrakulic, JassyRakov, A. V.Dreiseitl, AntonínRamadan, HazemDressler, FrankRamanathan, PalaniappanDrillien, RobertRamírez Soto, Max CarlosDroscha, CaseyRamsden, DavidDruszczyńska, MagdalenaRassow, JoachimDuarte, AidaRather, IrfanDuchemin, Jean-BernardRather, PhilipDufrasne, FrançoisRathnayake, Athri D.Duggan, JackieRay, SamriddhaDukic, MarinelaRazim, AgnieszkaDumetz, FranckRazzuoli, ElisabettaDumic, IgorRead, AndrewDumitrache, Mirabela OanaRecordon-Pinson, PatriciaDumitru, MIhaiRedfield, RosemaryDumoux, MaudReeves, Will K.Dunaj, JustynaRegunath, HariharanDunowska, MagdalenaReimão, Juliana QueroDurán-Poveda, ManuelRenieri, AlessandraDurham, Benjamin H.Repullés-Albelda, AigüesDutta, SanjuctaResende, TalitaDwidar, MohammedResta, LeonardoDybicz, MonikaRettich, FrantisekDykes, Gary A. A.Reynolds, ToddDylag, MariuszRibas Salvador, AlexisDzięgielewska, MagdalenaRibeiro, Rita Cláudia CardosoDziendzikowska, KatarzynaRicci, GiuseppeDzimianski, JohnRiccò, MatteoDzimira, StanisławRichi, PatriciaEastman, AlisonRickards, KarenEasy, RussellRidpath, JuliaEgan, SharonRiedel, ChristianeEibl, Martha M.Riehle, MichaelElaswad, AhmedRios Ocampo, W. AlfredoEl-Costa, HichamRipellino, PaoloEleftheriadis, TheodorosRitter, ManuelEl-Gazzar, Mohamed M.Riva, GiuseppeElguezabal, NataliaRizk, Mohamed AbdoElkheir, NatalieRobbins, MarthaElla, BhagyarajRobinson, KelsyEllervik, UlfRobinson, Mark W.Ellis, JohnRoby, Justin A.Elmahallawy, EhabRodrigo, LuisEl-Mayet, FouadRodrigues, ArmandaElshabrawy, Hatem A.Rodriguez, Sergio E.Elshamy, AbdelsamedRodríguez, William R.Emecen-Huja, PinarRodríguez-Jerez, José JuanEnose-Akahata, YoshimiRodriguez-Martinez, AlejandroEnosi Tuipulotu, DanielRodríguez-Rojas, AlexandroEpelde, FranciscoRóg, JoannaErickson, TimothyRojas, AliciaEscudero-Abarca, BlancaRojas-Martínez, CarmenEscudero-Pérez, BeatrizRojo, Maria AngelesEso, YujiRola-Łuszczak, MarzenaEsteban-Zubero, EduardoRollinson, DavidEstrada-Peña, AgustínRolo, JoanaEum, Seok-YongRomano, MarcoEverett, Helen E.Romano, Patricia SilviaFablet, ChristelleRosales, RubenFabrizi, FabrizioRose, TimothyFagundes, Nathlia Carolina FernandesRosenheim, Jay A.Falkinham III, Joseph O.Ross, Karen F.Falzon, LauraRossetti, Carlos A.Fan, LushengRossi, LucaFan, ShufangRossi, Shannan L.Fan, WenchunRostal, MelindaFan, Yi-ChinRota, PaulFarr, AlexRoura, XavierFarrell, HelenRousis, Nikolaos I.Fasciana, TeresaRoussel, AnneFasoula, VasiliaRouthu, Nanda KishoreFavennec, LoicRoux, Anne-LaureFavier, Anne-LaureRouzine, IgorFelicetti, TommasoRoy, DhruvajyotiFendrihan, SergiuRóżewicz, MarcinFeng, HuapengRubiola, SeleneFeola, AlessandroRubio-Tomás, TeresaFernandes, Luis Guilherme VirgílioRudrappa, MohanFernández Rodríguez, AmandaRueca, FabrizioFernández, Rita Sánchez-AndradeRugna, GianlucaFernández-Soto, PedroRuiz De Ybáñez, María RocíoFerra, BartłomiejRumbos, ChristosFerrante, PasqualeRumbou, ArtemisFerre, IgnacioRungelrath, ViktoriaFerreira, Ana CristinaRussini, ValeriaFerreira, MarianaRybczyńska-Tkaczyk, KamilaFerreira, NelsonRybka, Jakub DaliborFerreira, PedroSabeta, ClaudeFerrigo, DavideSabharwal, AnkitFerrins, LoriSabino-Santos, GilbertoFeuer, RalphSachithanandham, JaiprasathFiedler, TomasSádlová, JovanaField, MarkSah, PrakashFiester, SteveSahu, PranavFilardo, SimoneSaid, Elias A.Filippini, DavideSaito, Tais BerelliFinn, RobertSakamoto, Joyce M.Fioretti, AlessandraSakamoto, YuzuruFioretti, AlessandroSalamon, DominikaFischer, MelinaSalant, HaroldFlanagan, JohnSaleh, MonaFlannery, JohnSalina, ElenaFlorian, VasileSalje, JeanneFoglia Manzillo, ValentinaSallustio, FabioFöldvári, GáborSalpini, RominaFolliero, VeronicaSalvato, MariaFontaine, FlorenceSalzano, Anna MariaFoschi, Francesco G.Samardžija, MarkoFouladkhah, AliyarSam-Yellowe, TobiliFoutch, Brian K.Sánchez, IsraelFox, JulieSander, ValeriaFoxi, CiprianoSanders, Christopher J.Fraile, LorenzoSándor, Attila D.Franco, DomenicoSang, YongmingFranco-Paredes, CarlosSanna, GiuseppinaFratini, FedericaSantamaria, FlaviaFree, StephenSantamaria, OscarFreedman, AndrewSantamaría-Peláez, MirianFrey, CarolineSantella, BiagioFrías-De-León, María GuadalupeSanti, AnnalisaFriedman, Daniel Z. P.Santic, MarinaFrlan, RokSantolalla-Arnedo, IvánFry, Lindsay M.Santos, JeffersonFrye, Jonathan G.Santos, JenyFuchs, BethSantos, MariaFuchs, MarcSantos, Rita B.Fuehrer, Hans-PeterSantovito, GianfrancoFuentes, IsabelSantucciu, CinziaFuentes, Màrius V.Sapi, EvaFujita, AkikazuSaraiva, AuréliaFukuda, AkiraSarkar, SanjayFurin, Jennifer J.Sarmiento-Villamil, Jorge L.Fusco, Francesco MariaSassera, DavideFutoma-Kołoch, BożenaSatish, LakkakulaFux, RobertSato, IkuoGabriel, IwonaSato, Marcello OtakeGabryszewski, StanislawSato, TatsuoGaglio, GabriellaSato, TetsuoGajda, AnnaSauleda, SilviaGajdosik, Martina SrajerSauter-Louis, CarolaGalán-Puchades, María TeresaSavenkov, EugeneGaldiero, MassimilianoSavic, VladimirGałęcki, RemigiuszSayed, Ibrahim M.Gallardo, CarminaSchalkwyk, Antoinette VanGallego-Lopez, GinaSchatz, DaniellaGalletti, Maria F. B. M.Schifanella, LucaGallo, GaetanoSchijman, Alejandro GabrielGallo, MonicaSchildgen, OlivierGally, DavidSchlagloth, RolfGaluska, Sebastian PeterSchmid, Jeffrey RobertGálvez, RosaSchmidt, AnnikaGamage, Shantini D.Schmidt, MartinGambardella, ClaudioSchmitt-Keichinger, CorinneGamil, AmrSchmitz, BradleyGanges, LlilianneSchnee, ChristianeGanta, RomanSchnittger, LeonhardGao, XiaolongSchreiber, MichaelGarbowska, MonikaSchuberth, Hans J.García-Bujalance, SilviaSchulthess, BettinaGarcia-Bustos, Jose F.Schulz, KatjaGarcía-Gasca, Silvia AlejandraSchulz-Weidner, NellyGarcia-Rosado, EstherSchuppe Koistinen, InaGargala, GillesSchwarz, StefanGarijo Toledo, María MagdalenaSchweigkofler, WolfgangGarratt, Luke W.Scobie, LindaGarvy, Beth A.Seal, BruceGaudieri, SilvanaSebbane, FlorentGaudin, YvesSee, Pao TheenGaudreault, Natasha N.Seeber, PeterGaurivaud, PatriceSekiguchi, SatoshiGazzonis, Alessia LiberaSekse, CamillaGeary, Timothy G.Selim, Abdelfattah M.Geiger, StefanSeltsam, AxelGenchi, ClaudioSemih, EsinGenovese, FedericaSerrano, EmmanuelGenovese, Kenneth J.Sessa, RosaGeorge, SantoshSetz, ChristianGerken, Alison R.Seuberlich, TorstenGeyer, NathanielSeveri, GiulioGhafar, AbdulSevilla, EloísaGherman, Calin MirceaShah, JyotsnaGhiasi, HomayonShahin, KhalidGhosh, AmalenduShankar, SundareshGhosh, ArnabSharafi, HeidarGhosh, ArunavaSharafutdinov, IrshadGiaccone, ValerioSharma, ArvindGiacomelli, AndreaSharma, AtashiGiangaspero, AnnnunziataSharma, AtulGibson, AndySharma, PrashantGilbert, JeffreySharon, GalitGillespie, JosephSharp, Claire R.Ginger, MichaelSharshov, KirillGiorgio, SelmaShaw, DanaGirija, RamakrishnanShaw, HannahGirones, NuriaShchelkunov, Sergei NikolaevichGiuffrè, MauroShekhar Singh, ShashiGkimpas, GeorgiosShelite, Thomas RobertGlazer, ItamarShen, ShichenGlazunova, Olga A.Shiao, Shin-HongGniadek, AgnieszkaShih, Chien-MingGobin, IvanaShilev, StefanGoehring, LutzShim, DonghwanGoeser, FelixShimosato, TakeshiGolender, NatalyaShin, AesunGomez De León, Carmen T.Shin, Kwang-SooGómez López, PedroShin, MinkyoungGomez-Casado, CristinaShinozuka, YasunoriGomez-Lucia, EsperanzaShmakov, Sergey A.Gómez-Mejia, AlejandroShoesmith, EmilyGómez-Muñoz, María TeresaShor, ErikaGonciarz, WeronikaShoukry, NaglaaGong, LanShree, SonalGong, Yu-NongShridhar, SurekhaGongal, GyanendraShukla, NehaGonzález, Gloria M.Sichero, LauraGonzález, Jorge FranciscoSiddiqi, Mohammad KhursheedGonzález, Luis MiguelSiddiqui, ArifGonzalez, Miguel ToroSiddiqui, GhizalGonzález-Álvarez, Vicente HomeroSieg, MichaelGonzález-Barrio, DavidSigillo, LoredanaGonzalo, Diego GarciaSikorska, KatarzynaGordeev, IlyaSilas Fernandes, EtoGordon, Catherine A.Siltz, Lauren A.Gorgoglione, BartolomeoSilva, EdsonGorski, LisaSilva-Herzog, EugeniaGortázar, ChristianSim, ValerieGoto, YasuyukiSimecka, Jerry W.Gougoulis, Dimitris A.Simões, João Carlos CaetanoGould, Ernest A.Simonato, GiuliaGowans, EricSimonetti, Antonella FrancescaGowda, Ram C.Šimunović, KatarinaGraham, SheilaSinderewicz, EmiliaGraham-Brown, JohnSinger, StevenGranica, SebastianSingh, SukhvinderGrant, David C.Singh, VineetGrant, MatthewSinigaglia, AlessandroGrant, MichaelSioofy-Khojine, AmirbabakGrass, GregorSklaviadis, TheoGreco, GraziaSkliros, DimitriosGregorc, AlešSkoracki, MaciejGrgić, IvanaSkovgaard, OleGrier, Jennifer T.Skrypnik, Liubov N.Grippi, FrancescaSlonchak, AndriiGrodzki, MarcoSluder, Ann E.Groll, Andreas H.Šmajs, DavidGroschup, MartinSmodiš Škerl, Maja IvanaGross, Jane E.Šoba, BarbaraGrosso, FilipaSofia, MarinaGrouzi, ElisavetSojka, DanielGruber, ArthurSolana, José CarlosGruntmeir, JeffreySolano, Beatriz RamosGryschek, Ronaldo Cesar BorgesSolari, AldoGrzela, TomaszSoltys, KatarinaGuardone, LisaSommer, JuliaGuarino, CassandraSong, Hyun-OkGubert, CarolinaSoo Ko, KwanGuellil, MeriamSooryanarain, HariniGuerrero Espejo, AntonioSorgi, Carlos ArtérioGuerrero-Plata, AntonietaSpada, EvaGuignard, GaëtanSpergser, JoachimGuimarães, Maria AngelicaSpeziale, PietroGuinat, ClaireSpidlova, PetraGuito, JonathanSpiller, O. BradGunn, Bronwyn M.Spînu, MarinaGunn, John S.Špitalská, EvaGupta, KuldeepSplichal, IgorGupta, NimishSplichalova, AllaGupta, SeemaSquartini, AndreaGutierrez, Enriqueta GarciaSridhar, AdithyaGutiérrez, Silvina ElenaSriwastva, Mukesh KumarGutiérrez-Abejón, EduardoSt. Leger, Raymond J.Guttmacher, SallyStadejek, TomaszGuy, Rebecca A.Stadler, Liselott SvenssonGyörke, AdrianaStahl, FelixHaataja, SauliStanelle-Bertram, StephanieHabachi, MagdiStange, MadlenHade, MangeshStanković, IvanaHaelewaters, DannyStanton, RichardHaese, Nicole N.Stapleford, KennethHagen, FrickmannStasiak-Różańska, LidiaHagen, Ralf MatthiasStauft, CharlesHakimi, HassanStavropoulos, AndreasHall, RobynSteele, DuncanHalonen, Sandra K.Steinbach, FalkoHam, Jong HyunStevanović, VladimirHamaguchi, ShigetoStevens, Joanne M.Hanau, StefaniaSteyn, AngelaHancock, MeaghanStietz, SilvinaHaneke, EckartStigler Granados, PaulaHanif, ShumailaStilianakis, Nikolaos I.Hanson, LisaStingeni, LucaHarriott, LanaStranieri, AngelicaHarrison, Jessica J.Strati, KaterinaHarrod, RobertStromberg, ZacharyHarrus, ShimonStrommenger, BirgitHartley, CarolStuhl, Charles J.Hartmann, AntonStupica, DašaHarvey, WilliamSubash, SarguruHasan, Nabeeh A.Subedi, DineshHaslwanter, DeniseSubramaniam, SaravananHasni, IssamSugihara, KoheiHässig, MichaelSui, YongjunHattori, ToshioSulo, PavolHawman, DavidSultan, KhaledHayes, Sidney J.Sun, CaijunHe, BaoyeSun, XiangjieHeaton, Steven M.Sundaram, AppavuHedman, HaydenSundberg, Eric J.Hefferon, Kathleen LauraSundberg, Lotta-RiinaHeffron, AnnaSung, I-HsinHegazy, Wael A. H.Supronienė, SkaidrėHeidingsfeld, OlgaSurachetpong, WinHeinisch, JürgenSurewaard, Bas Gerardus J.Heinonen, Krista M.Suriano, FrancescoHelle, FrancoisSurleac, MariusHeller, MartinSuryadevara, VidyaniHenderson, AndrewSuttiprapa, SutasHeng, NicholasSvärd, StaffanHeng, XiaoŠvedienė, JurgitaHenriquez, FionaSwearingen, Kristian E.Hensen, LucaSykulev, YuriHepojoki, SatuSzczepanek, JoannaHerbold, JohnSzentgyörgyi, HajnalkaHeredero-Bermejo, IreneSzentiványi, TamaraHernandez-Alcoceba, RubenSzteyn, JoannaHerrera, GuadalupeSzymańska-Czerwińska, MonikaHerzog, CatherineTaank, VikasHeuer, HolgerTabain, IrenaHide, GeoffTaddei, SimoneHlaváčová, JanaTai, AndrewHo, Cheng-MawTai, William Chi-ShingHodny, ZdenekTajima, MotoshiHoffmanová, IvaTakano, TomomiHogardt, MichaelTakashima, EizoHojo, FuhitoTakhampunya, RatreeHonda-Okubo, YoshikazuTalbot, GuylaineHönig, VáclavTamba, MarcoHönigl, MartinTamura, TomokazuHorák, PetrTan, XuHorhat, DeliaTanaka, MasakazuHorhat, Florin GeorgeTandukar, SarmilaHorianopoulos, Linda C.Taniguchi, HiroakiHorii, YoichiroTassaneetrithep, BoonratnHornung, BastianTassis, PanagiotisHotea, IonelaTate, Jacqueline E.Hotta, AkitoyoTate, MichelleHouben, ManonTatullo, MarcoHouhamdi, LindaTaus, NaomiHove, PaidasheTavelli, AlessandroHrast, MartinaTaylor, MatthewHsu, Cheng-LungTeglas, MikeHsu, Wei-LiTellez-Lance, AngelaHu, GuangganTenenbaum, TobiasHu, JieTeramoto, TadahisaHu, JohnTerasaki, KaoriHu, QingThannesberger, JakobHu, YanmeiThekkiniath, JoseHubbard, MichelleTheologidis, IoannisHuber, Charlotte A.Thomas, OberhänsliHuck, OlivierThompson, AndrewHue, Erika S.Thumbi, S. M.Hughes, Holly R.Tibayrenc, MichelHugo, LeonTikhe, Chinmay V.Hukowska-Szematowicz, BeataTinelli, AntonellaHulse, LyndalTirosh-Levy, SharonHunfeld, Peter KlausTita, OvidiuHung, KennethTodorov, SvetoslavHuntley, Jason F.Todt, DanielHussain, HamidahTogawa, AtsushiHussain-Yusuf, HazizulTołkacz, KatarzynaHuttunen, MoonaTomasevic, IgorHyrina, AnastasiaTomazi, TiagoIannetti, LuigiTomczyk, ŁukaszIanni, AndreaTong, Wen HanIbrahim, Sulaiman S.Toranzo, Alicia E.Idda, Maria LauraTorremorell, MontserratIde, KazukiTorres-Perez, FernandoIgarashi, IkuoTóth, BrigittaIhle, Kate E.Toth, EricIkeh, MélanieTouzé, AntoineIliev, Asparouh I.Traversa, DonatoIm, Jung-GiTrdan, StanislavIngram, ThomasTrevisan, GiustoInic-Kanada, AleksandraTrinh, Kieu The LoanIntroini, ViolaTripathi, ArtiIsakova-Sivak, IrinaTripathi, JitendraIscaro, CarmenTroan, Brigid VictoriaIslam, Md. NazmulTroccoli, AlbertoIsoda, NorikazuTroxell, BryanIwagami, MoritoshiTruyen, UweIzdebska, Joanna N.Tsouloufi, Theodora K.Jääskeläinen, Anne J.Tsuboi, MayoJackson, Mary DarbyTsuchida, SachioJacobs, William RobertTsuchiya, HiroyukiJaenson, Thomas G. T.Tuchscherr, LorenaJaggavarapu, SiddharthTuffs, Stephen W.Jakab, EndreTumanov, Alexei V.Jamal, AtifTuno, NobukoJames, ArnoneTurco, JeniferJames, JoeTurska-Szewczuk, AnnaJamil, TariqTuttobene, Marisel RominaJános, SzenderákTyagi, SwatiJaramillo Ortiz, José ManuelTzora, AthinaJarausch, BarbaraTzortzakakis, EmmanouelJarocki, VeronicaÚbeda, CarlesJaswal, RajneeshUchida, LéoJavǔrková, Veronika GvoždíkováUeti, MassaroJeewon, RajeshUlibarri, GerardJenkins, AndrewUmapathi, Krishna KishoreJennings, ErnestUmber, MarieJensen, IngvillUnnasch, ThomasJeon, ByeonghwaUpton, Jason W.Jeong, Byung-HoonUraki, RyutaJezierska-Woźniak, KatarzynaUrbano, NicolettaJiang, JunyeUwiera, RichardJijie, RoxanaVadlejch, JaroslavJiménez-Ruiz, SaúlVaheri, AnttiJohn, EmilyVakharia, VikramJohnson, NicholasValdés, James J.Johnson, TammiValdezate, SylviaJolly, Emmitt R.Valencia-Cantero, EduardoJones, MarjorieValente, Nicola AlbertoJones, NicolaValentini, PieroJordan, AlbertValentin-Weigand, PeterJordan, HeatherValigurová, Andrea BardůnekJores, JörgValiuskaite, AlmaJung, EunkyungValle, Manuel RodriguezJung, SophieValles, Steven M.Kabue, Jean PierreVallès, XavierKachrimanidou, MelinaVan Der Hoek, LiaKaclikova, EvaVan Der Vossen, Jos M. B. M.Kadela-Tomanek, MonikaVan Domselaar, RobertKaden, ReneVan Engelenburg, SchuylerKahl, Barbara C.Van Haren, Simon D.Kahler, CharleneVan Leeuwen, Sander S.Kaidashev, Igor P.Van Meurs, Jan C.Kain, RenateVan Rhijn, NormanKalesh, KarunakaranVanlandingham, DanaKalligeros, MarkosVanrompay, Daisy C. G.Kalmár, ZsuzsaVáradi, GyörgyiKalyandurg, Pruthvi BalachandraVarallyay, EvaKamei, KaekoVarela, MonicaKaminuma, OsamuVascellari, MartaKamysz, ElżbietaVasileiou, Natalia G. C.Kanda, TatsuoVásquez-Velásquez, DavidKandel, Prem PrasadVazquez De Aldana, Beatriz RodriguezKang, Hyun MiVeerus, LiisaKang, WeiVelasco, LeonardoKanmani, PaulrajVelazquez-Salinas, LauroKanna Reddy, Hariprasada R.Veldman, KeesKant, SashiVelikova, TsvetelinaKanto, TakeshiVenkat, HeatherKao, CarolVerbyla, MatthewKar, ShantimoyVerghese, DhiranKaragiannakis, DimitriosVerhoeven, DavidKarakavuk, MuhammetVerissimo, PaulaKaralis, Vangelis D.Verra, FedericaKaramon, JacekVesa, Stefan CristianKaramonova, LudmilaVesikari, Timo H.Karanikola, ParaskeviVibin, JessyKaranis, PanagiotisVidana Mateo, BeatrizKaraś, MagdalenaViennet, ElvinaKarim, ShahidVigilato, Marco Antonio NatalKarniely, SharonVijay, Ajay KumarKarpiński, Tomasz M.Vijayakumar, SekarKarsten, Christina B.Vilela, AliceKarunakaran, EstherVilla, ChiaraKasiotis, KonstantinosVilla, LucaKasprzak, RobertVillari, SaraKathayat, DipakVillarruel-López, AngélicaKato, HirotomoVinogradov, MarkelKatsafadou, AngelikiVinuesa, TeresaKaushik, AshleshaViola, AngèleKaviani, MojtabaViranaicken, WildrissKavran, MihaelaVirovic-Jukic, LucijaKawaguchi, TakeshiVishnyakov, Innokentii E.Kawalek, AdamVitale, LucaKayali, GhaziVitalii, TimofeevKazimírová, MariaViuda-Martos, ManuelKe, HanzhongVizzini, PriyaKebriaei, RaziehVlahovic, TraceyKedrowicz, April A.Voellmy, IreneKeele, John W.Vogiatzaki, ChrysanthiKeeler, ShamusVon Bartheld, Christopher S.Kehn-Hall, KyleneVon Dobschuetz, SophieKeiffer, Timothy R.Von Fricken, MichaelKeisuke, SuganumaVooslo, WilnaKeller, LanceVotypka, JanKenna, Dervla T. D.Vu, LuanKerdsin, AnusakWada, HideoKern, PeterWadsworth, CristaKevill, JessicaWagner, Gabriel E.Keyel, Peter A.Wagner, SteffenKhafizov, Kamil F.Waheed, YasirKhalid, HattafWaiker, PrashantKhalil, Ahmed Mohamed AliWaissengrin, BarlizKhan, Aman UllahWajima, TakeakiKhan, KrishnenduWalczak, MarekKhatri, VishalWaliullah, SumyyaKhatun, AminaWalker, Robert J.Khodadadi, FatemehWalochnik, JuliaKichloo, AsimWang, Fun-InKidd, Stephen P.Wang, HuizhiKiefer-Meyer, Marie-ChristineWang, JiongKielian, MargaretWang, LihuaKieliszek, MarekWang, MinghangKiewra, DorotaWang, Robert Y.-L.Kim, Bum-JoonWang, Shao-HungKim, Hwa-JungWang, XinKim, Hwan KeunWang, Yung-ChihKim, KyoungtaeWang, ZhejunKim, Kyung HyunWańkowicz, PawełKim, MikyeongWarris, AdiliaKimura, HirokazuWąsowski, JacekKinkar, LiinaWassermann, MarionKinthada, LakshmanakumarWatier-Grillot, StéphanieKirkman, LauraWebb, CameronKishida, YutakaWebb, R. ClintonKiszewski, Anthony E.Węgrzyn, GrzegorzKitazumi, AiWehner, TimKitchen, MariaWein, AlexanderKittl, SonjaWeir, BevanKivistö, RauniWeir, DawnKizheva, YoanaWeissenböck, HerbertKjemtrup, AnneWenbin, ZhanKlafack, SandroWerszko, JoannaKlapper, PaulWest, JonathanKlewicka, ElzbietaWhelan, Adam O.Klimentova, JanaWhitworth, DeanneKline, Kimberly A.Wijnveld, MichielKlöhn, PeterWikel, Stephen K.Knowles, Donald P.Wilkens, LudwigKnox, CarolineWilkes, Rebecca P.Kobayashi, SotaWillcox, MarkKobayashi, TomokoWilliams, Richard A. J.Kochanowski, MaciejWilliams, Scott C.Kocisova, AlicaWilson, Alphus D.Kodym, PetrWilson, Danny W.Kokkinakis, Emmanouil N.Windshügel, BjörnKolawole, Abimbola O.Winston, Jenessa A.Kolb, PhilippWinter, StephanKollipara, AvinashWise, LynKołodziej, PrzemysławWiszniewska-Łaszczych, AgnieszkaKondo, HidehiroWitola, William HaroldKondru, NaveenWiwart, MarianKonradyova, VeronikaWojsyk-Banaszak, IrenaKontsevaya, IrinaWojtacka, JoannaKoos, BjörnWojtkowska, MałgorzataKopper, Jamie J.Wolfraim, LarryKopsidas, IoannisWood, BenKorzekwa, KamilaWortel, MeikeKorzeń, MarcinWoźniakowski, GrzegorzKoshizuka, TetsuoWright, Terry W.Kosoy, MichaelWronska, Anna KatarzynaKostoulias, XeniaWu, Fang-TzyKostov, GeorgiWu, Jack H. Z.Kostygov, Alexei YuWu, JianxuanKot, KarolinaWu, Terry H.Kotyza, PavelWu, YuntaoKovacs, RenatoWysocka-Mincewicz, MartaKowal, KrzysztofXi, ZhiyongKozačinski, LidijaXiao, LihuaKozieł, EdmundXiao, XiaKraiczy, PeterXie, HangKranz, AngelaXie, ShangzheKranzler, MarkusXie, ZhiqunKrawczyk, BeataXirogianni, AthanasiaKrawczynski, KamilXu, YiKrčmar, StjepanXue, GuangaiKretschmer, MatthiasYadav, DhananjayKrige, Anna-ShereeYadavalli, Tejabhiram TejabhiramKrivoruchko, AnastasiiaYamada, HiroyukiKroeker, AndreaYamanaka, AtsushiKrömker, VolkerYang, ChuanyuKruger, DetlevYang, KunKryukova, Natalia A.Yang, QianKrzysiak, Michał K.Yao, ChaoqunKubiak, KatarzynaYaparla, AmulyaKubiszewski-Jakubiak, SzymonYarullina, DinaKuczowski, MaciejYera, HélèneKuhs, Krystle A.Yim, Howard C. H.Kumar, AjayYoann, AiguKumar, HirdeshYoko, MatsuzakiKumar, SantoshYoo, Won GiKumar, VikashYordanova, IvetKunjeti, Sridhara G.Yoshimatsu, KumikoKuo, Chi-ChienYou, HongKuo, Ping-ChungYouk, SungsuKupnicka, PatrycjaYu, QingKusejko, KatharinaYuan, FangfengKusstatscher, PeterYuan, MengKuzmak, JacekYuan, ShuofengKuzmanovic, NemanjaYuen, Courtney MingmenKwak, DongmiYuill, ThomasKwiatek, AgnieszkaYumoto, HiromichiKwon, Seong YoungYurchenko, VyacheslavKwun, Hyun JinYuta, AstushiLaabei, MaisemZachou, KalliopiLabuda, RomanZahmanova, Gergana G.Lacour, Sandrine A.Žákovská, AlenaLafon, Marie-EdithZaleśny, GrzegorzLager, Kelly M.Zamoto-Niikura, AyaLahmers, KevinZarębska-Michaluk, DorotaLai, Michael M. C.Zavadilová, LudmilaLai, OlimpiaZawitkowska, JoannaŁakomy, PiotrZduńczyk, SławomirLam, YanZeman, PetrLamas, AlexandreZeng, MinxiangLambert, JohnZerbini, Francisco MuriloLamponi, StefaniaZerna, GemmaLandi, LuciaZerr, IngaLandolt, Gabriele A.Zhand, SarehLanger, Gitta JuttaZhang, MingLanghorst, Bradley W.Zhang, QiongLangridge, GemmaZhang, ShuoLantero, EstherZhang, TingLaroche, MaureenZhang, XiujuanLarson, CharlesZhao, BingchunLarzábal, MarianoZhao, LinboLasecka-Dykes, LidiaZhao, Pyrear SuhuiLaskowski, ZdzisławZhao, XiangliLaurimäe, TeiviZhao, Xue ZhiLauronen, JouniZheng, Du-PingLauzi, StefaniaZheng, MengmengLaval, KathlynZheng, XiaorongLavezzo, EnricoZhu, GuanLawrance, Matthew F.Zhu, WenjunLazarevic, IvanaZiarno, MałgorzataLe Breton, Yoann S.Zienius, DainiusLe Doaré, KirstyZientara, StefanLe Guern, Anne SophieZimowska, BeataLe, Hieu HuuZintl, AnnettaLean, Fabian Z. X.Żmudzki, JacekLeastro, Mikhail OliveiraZou, ZhongweiLeChevallier, MarkZubareva, Ekaterina V.Lecollinet, SylvieZumbrun, ElizabethLecureux, JonathanZuñiga, SoniaLedgerwood, Emily DesmetZurek, LudekLee, Chang-SeopZurita, AntonioLee, Hee IlZygner, WojciechLee, In-Ah


